# Results from *Ad Hoc* and Routinely Collected Data among Celiac Women with Infertility or Pregnancy Related Disorders: Italy, 2001–2011

**DOI:** 10.1155/2014/614269

**Published:** 2014-05-07

**Authors:** Francesca Fortunato, Domenico Martinelli, Rosa Prato, Biagio Pedalino

**Affiliations:** Department of Medical and Surgical Sciences, University of Foggia, 71121 Foggia, Italy

## Abstract

Celiac disease (CD) is a chronic autoimmune illness triggered by gluten consumption in genetically predisposed individuals. Worldwide, CD prevalence is approximately 1%. Several studies suggest a higher prevalence of undiagnosed CD in patients with infertility. We described reproductive disorders and assessed the frequency of hospital admissions for infertility among celiac women aged 15–49. We conducted two surveys enrolling a convenient sample of celiac women, residing in Apulia or in Basilicata (Italy). Moreover, we selected hospital discharge records (HDRs) of celiac women and women with an exemption for CD, and matched the lists with HDRs for reproductive disorders. In the surveys we included 91 celiac women; 61.5% of them reported menstrual cycle disorders. 47/91 reported at least one pregnancy and 70.2% of them reported problems during pregnancy. From the HDRs and the registry of exemption, we selected 4,070 women with CD; the proportion of women hospitalized for infertility was higher among celiac women than among resident women in childbearing age (1.2% versus 0.2%). Our findings highlight a higher prevalence of reproductive disorders among celiac women than in the general population suggesting that clinicians might consider testing for CD women presenting with pregnancy disorders or infertility.

## 1. Introduction

Celiac disease (CD) is a multifactorial chronic autoimmune systemic disease triggered by gluten consumption in genetically predisposed individuals [1].

Worldwide, the prevalence of CD in the general population is approximately 1%; female : male ratio is 2 : 1 [2–6]. In Europe, CD prevalence ranges between 0.5% in Germany and 2.4% in Finland [7]. In Italy, CD prevalence is between 0.55% and 1% [8].

Most frequently, CD presents with gastrointestinal symptoms; however, it may be also associated with extraintestinal signs and symptoms and in women with reproductive disorders [9, 10]. CD has been associated with recurrent spontaneous abortion [10–20], intrauterine growth restriction, preterm delivery and low-birth weight [11–14, 16–27], infertility, delayed menarche, early menopause, and stillbirth [11, 28–32]. The risk of multiple abortions is 8 to 9 times higher in women with untreated CD than among treated patients [15]. CD has also been associated with gynecologic disorders such as amenorrhea [10, 23, 31, 33]. These may even be the only presenting features and are considered atypical clinical forms of CD [11, 13–15, 34].

Several studies suggest a higher prevalence of undiagnosed CD in patients with infertility [10, 12, 35–40]. In Europe, the prevalence of CD among infertile women varies between 4% and 8% [15, 30, 35, 36, 41–43].

We described the type and frequency of reproductive disorders in a sample of celiac women recruited from two Italian regions and assessed the frequency of hospital admissions for fertility related problems among celiac women resident in Apulia.

## 2. Methods

### 2.1. Surveys in Two Different Italian Regions

We enrolled a convenient sample of celiac women, diagnosed by a specialist, aged 15 to 49 years and residing either in Apulia or in Basilicata (two neighboring regions in the south of Italy) presenting a similar distribution of the population by age and sex. Recruitment of subjects was performed among women attending a conference of the Italian Association of Celiac Disease (AIC), held in April 2008 and among women entering a shop selling gluten-free products during the month of May 2008 in Apulia and in Basilicata, respectively.

We obtained informed consent to participate in the study and administered a standardized questionnaire aiming at investigating (as main outcomes) history of menstrual cycle disorders, history of problems during pregnancy, and history of infertility. Data were anonymized in compliance with the privacy law in force [44].

We used the ANOVA test for continuous variables (gestation week, birth weight, and breastfeeding duration) with normal distribution, using as independent variable the gluten-free diet (GFD) during pregnancy and during breastfeeding, respectively; in alternative we used the nonparametric Kruskal-Wallis test. To assess the distribution of the variables, we used Bartlett's test. We assessed the possible associations among the explored variables by defining double-entry contingency tables and calculating Odds Ratio (OR) with 95% CIs. We analyzed data using Stata v.12 software.

### 2.2. Population Analysis from Available Registries

**Figure 1:**
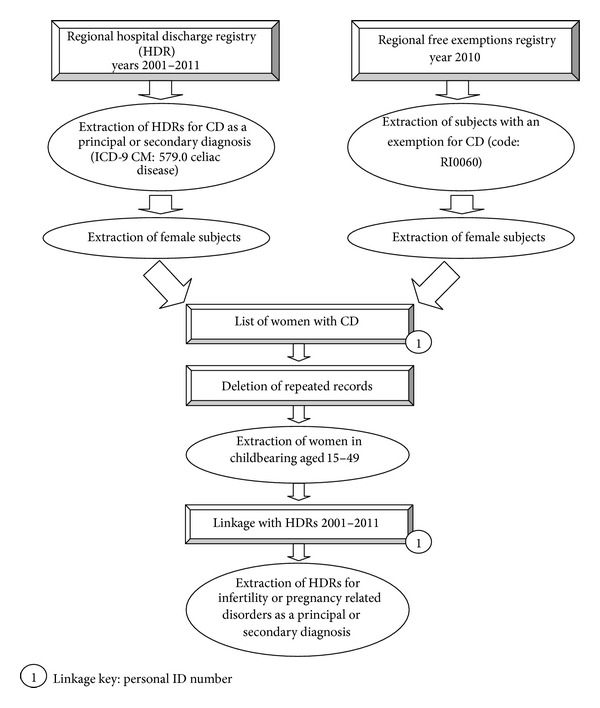
Algorithm of the population analysis from available registries. Apulia, years 2001–2011.

In order to obtain an exhaustive list of celiac women in the Apulia Region we used two sources: (1) the regional hospital discharge registry and (2) the users free exemptions registry. The latter contains information on chronic patients that do not pay medical consultations for their specific chronic disease; each disease is identified by a specific and unique code [45, 46]. From the regional hospital discharge records, from year 2001 to 2011, we extracted women resident in Apulia who had been discharged from the hospital with CD as either main or secondary diagnosis (ICD-9 CM: 579.0* celiac disease*). From the users free exemptions registry, year 2010, we extracted women with an exemption code for CD (code: RI0060). Subsequently, we matched (linkage key: personal ID number—unique number given at birth to each new born in Italy; the use of the ID number prevents having doubles among the records, e.g., one discharge record equal to one woman) the obtained list of celiac women with all available years of the hospital discharge registry (years 2001–2011) selecting all discharges with one or many of the following diagnosis or procedure for infertility or pregnancy related disorders (Figure 1).  Table 1 shows the ICD9-CM and exemption codes used for the selection of the study population.

We compared the proportions of hospitalization for the considered pregnancy related disorders (Table 1) between celiac women and the remaining women resident in Apulia during the years 2001–2011 over the total number of women with at least one pregnancy (celiac and not celiac, resp.). Moreover, we compared the proportion of hospitalization for infertility in celiac women with the same proportion in the general population over the total number of celiac women, as calculated in Figure 1, and the total of women resident in Apulia, respectively.

To evaluate potential associations across the variables explored double-entry contingency tables (2 × 2) were defined and the chi-square (×2) value determined by considering* P* values <0.05 as significant.

## 3. Results

### 3.1. Surveys in Two Different Italian Regions

In the two Italian regions, 91 celiac women were interviewed (62 from Apulia and 29 from Basilicata). Median age was 32 ± 9.6 (range 17–49 years). The median age at diagnosis was 25 ± 13.2 (range 1–47 years). Diagnosis of CD was made by a medical specialist in 100% of cases (*n* = 91): the serum levels of antigliadin antibodies (AGA) was measured in 59.3% (*n* = 54), antiendomysial antibodies (AEA) in 57.1% (*n* = 52), and antitransglutaminase (tTG IgA) in 37.4% (*n* = 34). Biopsy of the small intestine was performed in 47.2% (*n* = 43) of the interviewed celiac women. Most women (*n* = 65, 71.4%) reported gastrointestinal symptoms as presenting symptom of CD. 56 cases (61.5%) reported bloated stomach as onset symptom (Table 2).

Almost all of the celiac women (*n* = 90, 98.9%) had been on a GFD for 8.1 ± 7.6 years (95% CI: 6.5–9.7 years) on average. The median age at menarche was 13 ± 1.5 years. Most celiac women (*n* = 56, 61.5%) reported a past history of at least one menstrual cycle disorder (Table 3).

Among the celiac women with a history of menstrual cycle disorders, in 69.6% (*n* = 39) of cases the diagnosis of CD was done after the onset of gynecologic symptoms; in 58.9% (*n* = 33) before or at the same time of the signs and symptoms of the CD; 66% (*n* = 37) of women were not on a GFD when the symptoms occurred.

At the time of the interview, 55.3% (*n* = 31) of celiac women reported experiencing menstrual cycle disorders; among them, 67.7% and 32.3% reported to suffer rarely and frequently, respectively.

Of the interviewed women, 52.2% (*n* = 47) had one or more pregnancies. Overall, the participants reported a total of 99 pregnancies (average number of pregnancies 2.1 ± 0.9); among them, 80 were to full term pregnancies and 19 miscarriages (six women reported 1 miscarriage each, two reported two miscarriages, and three of them reported three miscarriages each). Overall, 70.2% (*n* = 33) of celiac women reported having problems during pregnancy.

In 44.4% (*n* = 44) of pregnancies women reported a hemorrhage in early pregnancy, in 31.3% (*n* = 31) severe anaemia, in 13.1% (*n* = 13) gestational hypertension and placental abruption, in 5% (*n* = 5) uterine hyperkinesia, and in 4% (*n* = 4) a deficit of intrauterine growth.

In 74.5% (*n* = 35) and in 21.3% (*n* = 10) of the women reporting one or more pregnancies, the diagnosis of CD was made after and before their pregnancy, respectively. Two women did not answer the question. Moreover, 31.9% (*n* = 15) reported that signs and symptoms attributable to CD occurred after the pregnancy, 29.8% (*n* = 14) before, and 25.5% (*n* = 12) at the same time. For six women, the temporal sequence between signs or symptoms of CD and pregnancy was not reported.

Disorders in pregnancy were more frequent in mothers not on a GFD (OR: 0.34; 95% CI: 0.14–0.85; *P* = 0.0192) (Table 4).

Although the difference was not statistically significant, the duration of pregnancy was shorter among those who were not on a GFD than among those who were following the diet (37.2 ± 3.2 versus 38 ± 2.3 weeks;* F*: 1.0058, *P* = 0.31909).

The average weight at birth of children born from celiac women was 2782.2 ± 609.5 grams. Again, although the difference was not significant, the weight at birth was lower in children born from mothers who were not on a GFD than among those who were on a diet (2732.5 ± 609 versus 2882.9 ± 629 grams;* F*: 0.99148, *P* = 0.32254).

The duration of breastfeeding was similar between those celiac women who were on a GFD during breastfeeding and those who were not on that diet (165.6 ± 99.7 versus 163.3 ± 103.6 days;* F*: 0.00559, *P* = 0.94067).

Eleven (12.1%) celiac women reported attempts to have a child unsuccessfully.

### 3.2. Currently Collected Data

In Apulia, 11,590 hospital discharge records for CD (main or secondary diagnosis) were reported between 2001 and 2011; among them, 7,882 (68%) were women. From the users free exemptions registry, we extracted 6,765 individuals with an exemption for CD; among them, 4,776 (70.6%) were women.

Adding the results obtained from the two databases, a total of 6,530 records of women with CD with either hospital discharge or an exemption for CD or both were identified. Among them, we selected women who were 15–49 years old and obtained a list of 4,070 women in childbearing age with either main or secondary hospital discharge diagnosis of, and exemption for, CD. We then matched this list with the available data from the hospital discharge registry (years 2001–2011).

Among the 4,070 celiac women, 51 (1.2%) were discharged from hospital with main or secondary diagnosis of sterility, and 20 (0.5%) reported having undergone an in vitro fertilization (IVF).

Overall, 27.3% (*n* = 1,113) of celiac women reported at least one pregnancy, and 1,697 pregnancies (average of 1.5 ± 0.7 pregnancy per celiac woman versus 1.75 ± 10.2 in the general population) were recorded.

Between 2001 and 2011, the proportion of hospitalization among celiac women was higher than among resident women in childbearing age for hemorrhage in early pregnancy, deficit of intrauterine growth and anemia, and we found similar findings for spontaneous abortion, gestational hypertension, and placental abruption. Furthermore, the proportion of women hospitalized for fertility related disorders was also higher among celiac women than among resident women in childbearing age (1.2% versus 0.2%) (Table 5).

## 4. Discussion

In a low proportion of women participating in the surveys diagnosis was made using the tTG IgA, whereas the proportion of those tested by AGA antibody was higher. However, at the time of the surveys, tTG IgA measurement was not yet recommended in the guidelines as the first choice test for patients with a suspect of CD [47, 48].

In line with most studies, women in our study reported gastrointestinal complaints as onset of CD [10, 42]. In detail, the prevalence of women participating in the surveys reporting fertility problems was higher than that reported from other studies (12.1% versus 4–8%) [15, 30, 35, 36, 41–43], whereas it was lower in the analysis of currently collected data (1.2%), likely because infertility might be managed by a specialist and may not require hospitalization. In addition, the proportion of problems during pregnancy was higher among women recruited for the surveys than in the currently collected data analysis. The average number of pregnancies was lower in the currently collected data analysis than in the surveys (1.5 versus 2.1).

Several studies showed the association between CD and infertility and pregnancy disorders [10–27, 35–40]. Our findings show that duration of pregnancy was shorter among women who were not on GDF than among those who were and that the neonatal birth weight was lower among children born from women who were not on GFD, although we could not highlight a significant difference. Another study conducted in Northern Italy on a large sample of women (*n* = 868) who gave birth to premature and/or small for gestational age (SGA) children provided consistent evidence that the prevalence of undiagnosed CD in mothers of SGA infants is higher than in the general population [27]. When comparing celiac women and women in childbearing age resident in Apulia the number of hemorrhage in early pregnancy, deficit of intrauterine growth, anaemia, and infertility was higher among celiac women. In light of the underdiagnosis of the CD (hence, the number of celiac women is underestimated) the difference could be even larger in the two groups. The number of spontaneous abortions, gestational hypertensions, and placental abruptions was, however, similar in the two groups. This difference might be due to nutritional deficiencies caused by malabsorption that occurs in untreated CD [49].

Our study might have some limitations. A possible weakness of the study design is that only 47% of the women with CD referred to have undergone small intestinal biopsy when they were asked for the diagnosis. Furthermore, the enrolment of celiac women for the surveys in the two regions may be biased by their attendance to the conference and to the shop, respectively. This may have led to an under- or overestimation of our findings. Moreover, the use of the hospital discharge diagnoses as source of information on hospitalized celiac women might either under- or overestimate the number of outcomes as it relies on the review of the discharge forms (where, i.e., diagnostic tests performed during hospitalization are not reported) rather than on the review of the medical records of celiac women. We thus could neither investigate further any risk factors and highlight any association between fertility/pregnancy disorders and celiac disease nor apply any clinical case definitions to our cases as we relied on the ICD-9 codes used at discharge by the hospital specialist. In order to limit the underestimation of the outcomes related to the CD among hospitalized women, we used the users free exemptions registry to capture also celiac women who had not been admitted to hospital.

Our findings, consistently with other published results, suggest that clinicians might consider testing for CD, as part of the differential diagnosis, women presenting with pregnancy disorders or infertility [6, 10, 12, 23, 27]. Further studies should, however, be conducted to support the health economic aspects of this decision.

## Figures and Tables

**Table 1:** ICD9-CM of diagnosis or procedures and exemption codes considered in the population analysis, years 2001–2011.

Diagnosis or procedures	Codes ICD9-CM
Celiac disease	**579.0**
Main or secondary diagnosis of sterility	
Infertility, female	628
Procedure of artificial insemination among celiac women	
Artificial insemination	V26.1, 69.92
Aspiration of ovary	65.91
Other operations on cervix and uterus	69.99
Main or secondary diagnosis of pregnancy related disorders among celiac women	
Spontaneous abortion	634
Hemorrhage in early pregnancy	640
Hypertension complicating pregnancy, childbirth, and the puerperium	642
Premature separation of placenta	641.2
Poor fetal grow	656.5
Anemia	648.2

Exemption	Code

Celiac disease	RI0060

**Table 2:** Type and frequency of celiac disease symptoms of onset, Italy, 2008.

Symptom	*n *	%
Bloated stomach	56	61.5
Anemia	48	52.7
Weight loss	44	48.3
Diarrhea	36	39.6
Vomiting	18	19.8
Other symptoms/signs	31	34.1

**Table 3:** Type and frequency of menstrual cycle disorders, Italy, 2008.

Menstrual cycle disorder	*N *	%
Premenstrual syndrome	40	71.4
Dysmenorrhea	37	66.1
Hypomenorrhea	22	39.3
Oligomenorrhea	20	35.7
Amenorrhea	15	26.8
Menometrorrhagia	13	23.2
Metrorrhagia	11	19.6
Polymenorrhea	8	14.3

**Table 4:** Association between disorders in pregnancy and being on a GFD, Italy, 2008.

Pregnancy disorder	GFD			OR (95% Confidence Interval)
	Yes	No	Total	
Yes	15	53	68	0.34 (0.14–0.85)
No	14	17	31	
Total	**29**	**70**	**99**	

**Table 5:** Hospitalization among celiac women and women from the general population (both aged 15–49 years), by condition/complication, years 2001–2011.

Condition/complication	*N* (%) celiac women	*N* (%) women general population	*χ* ^2^	*P* value
Hemorrhage in early pregnancy	155 (13.9)	45,677 (10.8)	8.7	<0.01
Spontaneous abortion	58 (5.2)	22,284 (5.3)	0.01	>0.05
Hypertension complicating pregnancy, childbirth, and the puerperium	35 (3.1)	15,795 (3.7)	1.02	>0.05
Placental abruption	7 (0.6)	2,838 (0.7)	0.03	>0.05
Deficit of intrauterine growth	40 (3.6)	8,476 (2.0)	13.4	<0.01
Anaemia	55 (4.9)	11,589 (2.7)	18.6	<0.01
*Infertility *	* 51 (1*.*2) *	*19,765 (0*.*2) *	*10*.*6 *	*<0*.*01 *
